# Ensemble percepts of colored targets among distractors are influenced by hue similarity, not categorical identity

**DOI:** 10.1167/jov.24.11.12

**Published:** 2024-10-16

**Authors:** Lari S. Virtanen, Toni P. Saarela, Maria Olkkonen

**Affiliations:** 1 Department of Psychology, Faculty of Medicine, University of Helsinki, Helsinki, Finland

**Keywords:** color, ensemble perception, psychophysics, categorization, summary statistics

## Abstract

Color can be used to group similar elements, and ensemble percepts of color can be formed for such groups. In real-life settings, however, elements of similar color are often spatially interspersed among other elements and seen against a background. Forming an ensemble percept of these elements would require the segmentation of the correct color signals for integration. Can the human visual system do this? We examined whether observers can extract the ensemble mean hue from a target hue distribution among distractors and whether a color category boundary between target and distractor hues facilitates ensemble hue formation. Observers were able to selectively judge the target ensemble mean hue, but the presence of distractor hues added noise to the ensemble estimates and caused perceptual biases. The more similar the distractor hues were to the target hues, the noisier the estimates became, possibly reflecting incomplete or inaccurate segmentation of the two hue ensembles. Asymmetries between nominally equidistant distractors and substantial individual variability, however, point to additional factors beyond simple mixing of target and distractor distributions. Finally, we found no evidence for categorical facilitation in selective ensemble hue formation.

## Introduction

**Figure 1.**
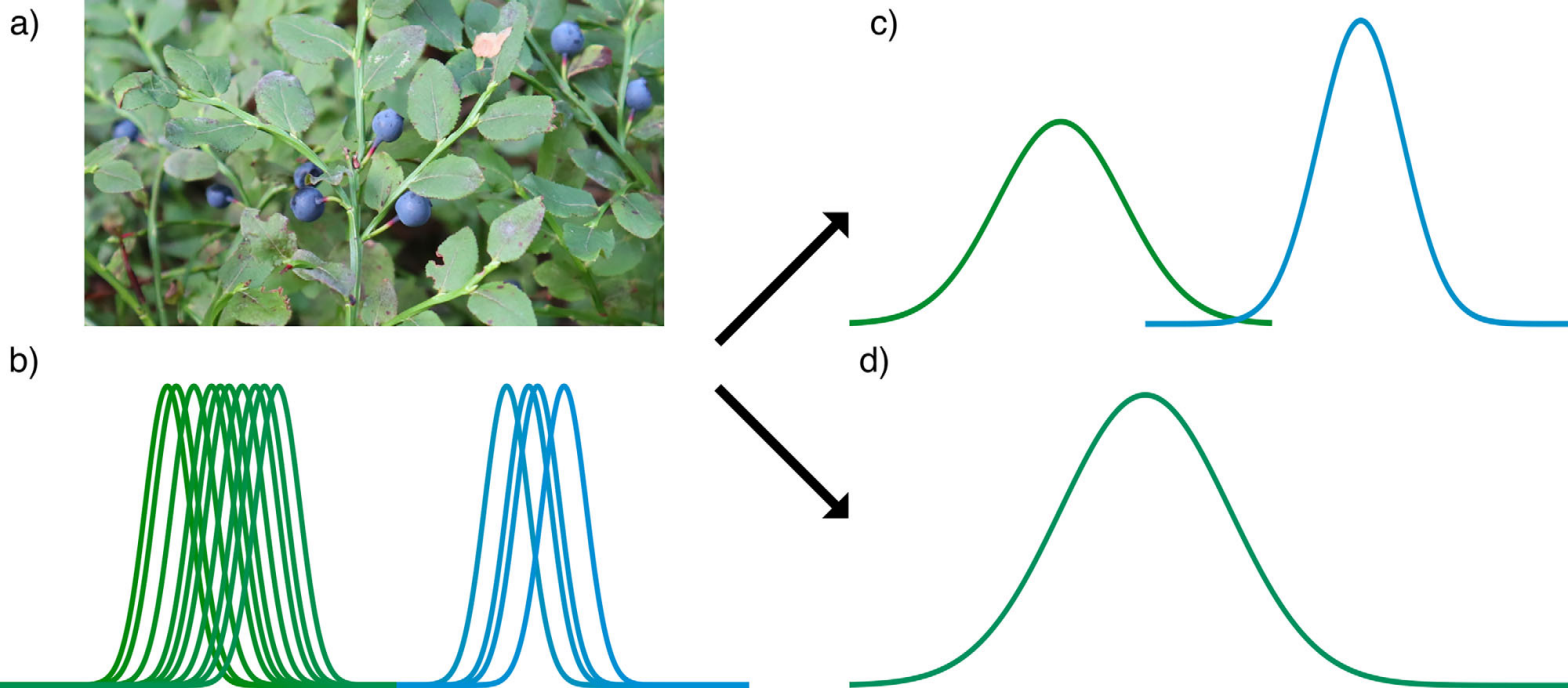
Processing of color distributions in a scene. (a) Objects of a certain color (blueberries) may be spatially interspersed with other colors in a scene (foliage). (b) A simplified illustration of different color signals within a scene, with each bell curve representing one noisy color signal in the hue dimension. Combining the signals into an estimate of the mean hue, the visual system might either (c) form an internal representation with separate peaks for the different color distributions or (d) combine all signals into an estimate of the grand mean.

Color information provides important, unique cues for segmenting and parsing the visual environment. For example, color provides advantages in visual search (D’Zmura, 1991), object recognition (Gegenfurtner & Rieger, 2000; Tanaka & Presnell, 1999), memory (Gegenfurtner & Rieger, 2000; Wichmann, Sharpe, & Gegenfurtner, 2002), and determining three-dimensional shape (Kingdom, 2003). Color also supports scene segmentation by edges (Kingdom, 2003; Tyler & Solomon, 2019) and textures (Goda & Fujii, 2001; Li & Lennie, 1997; Li & Lennie, 2001; Saarela & Landy, 2012) and enhances luminance-based edge perception (Hansen & Gegenfurtner, 2009). Surfaces of natural objects often exhibit a dominant hue, and any variation in hue tends to be in a limited range (for example, with fruits and vegetables, Ennis, Schiller, Toscani, & Gegenfurtner, 2018). Hue information across the scene can thus effectively guide rapid detection of specific objects without selective attention (D’Zmura, 1991; Wolfe, 2021), and separate color distributions might provide an effective cue in categorizing related groups of objects across the visual scene (Utochkin, 2015). Figure 1 illustrates a color selection task for finding groups of ripe blueberries hiding among green foliage. The blueberries and leaves give rise to noisy color signals distributed around their typical hues (Figure 1b). For an accurate estimate of the average blueberry color, the visual system should average signals separately for the blueberries and leaves (Figure 1c). However, the visual system may sometimes confound spatially intermixed color signals when estimating an average (Figure 1d). Here we examine whether and in which conditions the visual system can form separate estimates of the average color of spatially intermixed color distributions.

Ensemble perception refers to the visual system’s ability to swiftly and automatically extract some basic statistical properties like mean, range, and variance from a group of stimuli and has been linked to the perception of gist in a visual scene (Alvarez, 2011; Whitney & Yamanashi Leib, 2018). Ensemble percepts can be formed for a variety of stimulus features, such as size (Ariely, 2001; Chong & Treisman, 2003), orientation (Dakin & Watt, 1997; Parkes, Lund, Angelucci, Solomon, & Morgan, 2001), facial expressions (Haberman & Whitney, 2007; Haberman & Whitney, 2009), and even semantic categories (Khayat, Fusi, & Hochstein, 2021; Khayat & Hochstein, 2019). Generally, observers can only report low-order statistical properties from ensemble percepts (e.g., mean, range), but careful investigation has revealed a more nuanced internal representation. Observers seem to exclude or downweight deviant items of the ensemble in “robust averaging” (De Gardelle & Summerfield, 2011; Haberman & Whitney, 2010, but see Juni, Singh, & Maloney, 2010), reflected in ensemble averages being biased toward the mode of a skewed distribution (Iakovlev & Utochkin, 2023; Teng, Li, & Zhang, 2021, but see Virtanen, Olkkonen, & Saarela, 2020). Besides the mean, percepts show adaptation to ensemble variance (Jeong & Chong, 2020; Maule & Franklin, 2020; Norman, Heywood, & Kentridge, 2015) and encoding of the distribution shape (Chetverikov, Campana, & Kristjánsson, 2017; Hansmann-Roth, Kristjánsson, Whitney, & Chetverikov, 2021; Kim & Chong, 2020). These effects can be captured by accounts based on population coding of a stimulus feature (Iakovlev & Utochkin, 2023; Utochkin, Choi, & Chong, 2023).

Ensemble percepts of color have mostly been studied for hue distributions with constant chroma and lightness. Average hue percepts can be formed for fairly wide hue distributions, but the ability eventually breaks down for large hue variances (Maule & Franklin, 2015). As hue is defined as an angle in color space, this makes intuitive sense: For hues on opposite sides of the hue circle, a circular mean is not defined. The average color in Cartesian coordinates in this case is achromatic, but such averaging over complementary hues is not seen in observers’ summary estimates (Rajendran, Maule, Franklin, & Webster, 2021). With 16-element ensembles consisting of four distinct hues, Maule and Franklin (2016) found that information from just two items of the ensemble was enough to account for observer performance. With ensembles drawn from continuous hue distributions instead, observers were utilizing a large proportion of available information in their mean estimates (Virtanen, Olkkonen, & Saarela, 2020), in line with ensemble perception studies generally (Whitney & Yamanashi Leib, 2018). These studies have asked observers to average all chromatic elements on the display, and whether it is plausible to segment and only average elements of a specific hue distribution remains an open question.

Observers are able to form ensemble percepts of one stimulus feature over a subset of stimulus elements when another stimulus feature is provided as a segmentation cue (e.g., Halberda, Sires, & Feigenson, 2006; Im & Chong, 2014; Khvostov, Iakovlev, Wolfe, & Utochkin, 2024; Utochkin & Vostrikov, 2017). In these cases, segmentation is best driven by separable, basic stimulus features and less effectively by feature conjunctions (Khvostov et al., 2024). This “segmentability” of feature distributions is found to facilitate visual search of size and orientation (Utochkin & Yurevich, 2016), and discrimination of feature conjunctions (correlation of length and orientation) swiftly and across the whole stimulus display (Utochkin, Khvostov, & Stakina, 2018). Further, observers seem to be able to form an appropriate internal model of the distribution shape for correct segmentation (Chetverikov, Campana, & Kristjánsson, 2020). Some studies, however, have found that the ensemble percepts are biased toward the grand mean of all stimulus elements (Im, Tiurina, & Utochkin, 2021; Oriet & Brand, 2013). Here we directly examine the perceptual ensemble representation of the stimulus feature used as a segmentation cue. Our first aim is to investigate whether observers are able to extract an accurate and precise ensemble hue percept for target stimuli spatially interspersed with distractor stimuli and how that depends on the hue distance between targets and distractors.

Mental color representations can be both continuous and categorical, with the categorical nature more pronounced in tasks involving visual working memory (e.g., Bae, Olkkonen, Allred, & Flombaum, 2015). Categorical distinctions of hue distributions could thus hold significance for segmentation and categorization of groups of objects across the visual scene. Although there are substantial individual and language-related differences in the structure of categorical color space, these different patterns are well described by a few universal motifs (Lindsey & Brown, 2009; Lindsey & Brown, 2021). Categorical representations of color can influence color perception by, for example, enhancing color discrimination at category boundaries, a phenomenon often referred to as categorical facilitation (see Witzel & Gegenfurtner, 2018, for a review). Categorical facilitation could influence ensemble segmentation by enhancing sensory discrimination between different distributions but also by providing a salient criterion for segmentation. As ensemble percepts can be formed for higher-level stimuli such as facial expressions (Haberman & Whitney, 2007; Haberman & Whitney, 2009) and semantic categories (Khayat, Fusi, & Hochstein, 2021; Khayat & Hochstein, 2019), ensemble perception is not strictly limited to basic stimulus features. Thus, segmentation may also not be limited to basic stimulus features but reflect learned categorizations or feature relationships. Our second aim is to study whether color categories facilitate ensemble segmentation by hue.

Here, we investigate whether the visual system can selectively extract and process signals from spatially intermixed hue distributions when hue is the only cue to item membership. Many different stimulus features can collectively guide the perceptual organization of a visual scene (Wagemans et al., 2012); to rule out the effects of other visual features and focus only on the ensemble representation of hue, we employed simple ensembles of circular hue elements. Across two experiments, we measured precision and bias in an ensemble color comparison task while varying the distance and direction in hue angle between target and distractor distributions (Experiment 1) and the location of the target and distractor distributions relative to a color category boundary (Experiment 2). In summary, we found that the visual system can selectively form ensemble percepts of the target hue distribution but that the percept is less precise and biased toward distractor hues when target and distractor hues are more similar. We found no effect associated with color category boundaries.

## Methods

### Observers

Thirty-six observers took part in the study (16 in Experiment 1 and 20 in Experiment 2). In Experiment 1, five of the observers were students carrying out the measurements for a research practical course, and one observer was the first author of this article. All other observers were naive to the purpose of the study. Out of the 36 observers, 25 were female, and observer age ranged from 18 to 49 with a median of 22. All observers reported having either normal or corrected-to-normal visual acuity, and color vision was screened using Ishihara color plates (Ishihara, 1987). Observers gave informed consent and received either course credit or gift certificates as compensation for their time. The study protocol follows the Declaration of Helsinki, and methods were approved by the University of Helsinki Ethical Review Board in Humanities and Social and Behavioural Sciences.

### Apparatus

Measurements were done in a darkened laboratory room. Experiments were run with custom code in MATLAB (Version 2016b, Build 9.1.0.441655) with Psychophysics Toolbox Version 3 (Brainard, 1997; Kleiner, Brainard, & Pelli, 2007; Pelli, 1997) on an HP Z230 Desktop PC. Stimuli were displayed on a 23-inch ViewPixx 3D Lite display, controlled by an Nvidia Quadro K620 video card. The display primary spectra and gamma functions were measured using a SpectraScan PR-655 spectroradiometer, and these measurements were used to calibrate color rendering and to linearize display luminance. The maximum luminance of the display was 250 cd/m^2^, and the white point was set to be metameric to D65. The display output had 10-bit accuracy in intensity per channel with a 100 Hz refresh rate. The display pixel resolution was 1,920 × 1,080, but due to the high-bit-depth color mode halving horizontal resolution in the image, the effective resolution was 960 × 1,080. Viewing distance was kept constant at 100 cm using a chin rest, and observers used a regular keyboard to respond.

### Stimuli

All stimulus colors were defined in CIELUV color space, using LCh (lightness, chroma, and hue) cylindrical coordinates. Stimulus L and C coordinates were held constant at 70. All stimulus colors were thus drawn from a hue circle with radius 70 in CIELUV color space, and later mentions of hue circle refer to the one used here. A chromatic noise background was constantly displayed on the screen. Chromatic noise was produced by dividing the screen into 10-by-10-pixel squares, and a random color was drawn for each square in LCh-coordinates with ranges for L: 62.5–67.5, C: 0–5, and h: 0–360. Average background luminance was 35 cd/m^2^.

**Figure 2.**
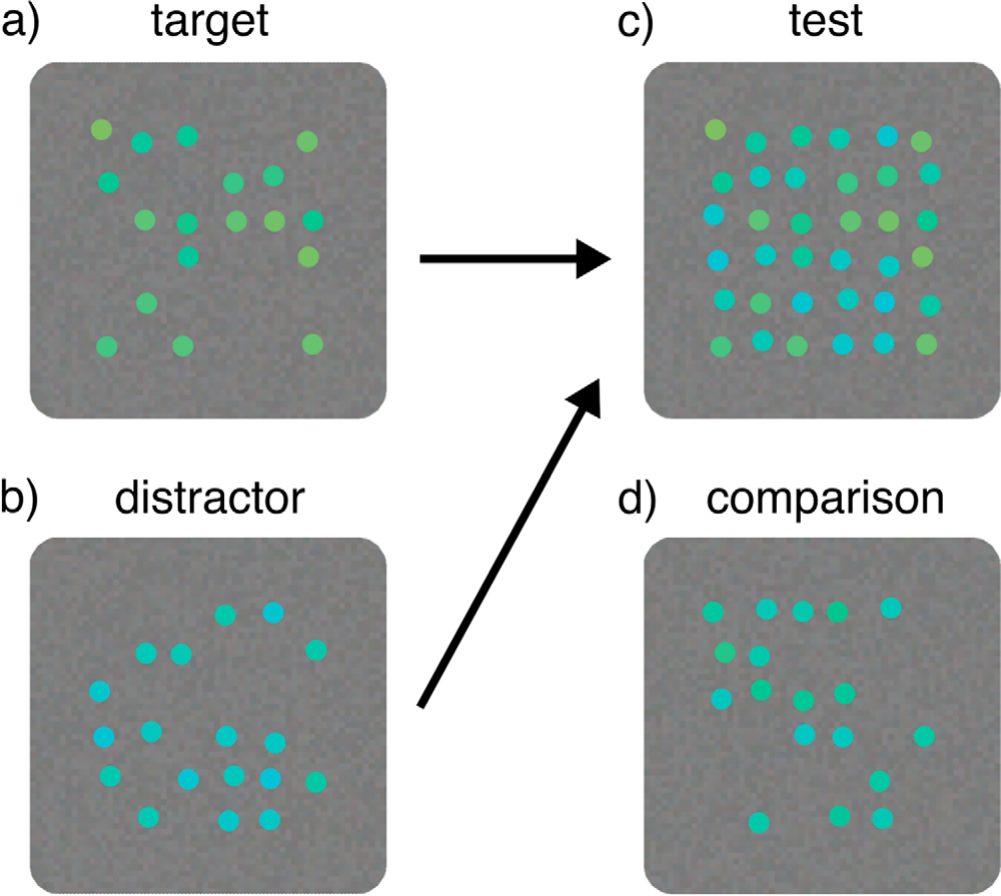
Example stimuli. The target (a) and comparison (d) ensembles were made of 18 colored dots. In some experimental conditions, a distractor ensemble (b) filled the empty space in the target ensemble to produce the test stimulus (c).

For the main experiments, a single ensemble consisted of 18 colored dots sized 0.5° in visual angle. The dots were placed randomly on a 6-by-6 grid in the middle of the screen, with an average center-to-center spacing of 1° in visual angle between adjacent dots (see, e.g., Figure 2a). For each trial, the location of the grid was jittered for a maximum distance of 1 degree in visual angle in a random direction. Additionally, the location of each individual dot on the grid was jittered for a maximum of 0.2° in visual angle in a random direction. Target (Figure 2a) and comparison (Figure 2d) were the task-related ensembles that observers were asked to compare in the experiments. In some experimental conditions, a task-unrelated distractor set of 18 colored dots (Figure 2b) filled the remaining empty cells in the target stimulus grid to form the complete test stimulus (Figure 2c).

For the stimulus dot colors, the hue of the dots was varied by drawing hue values from a uniform distribution around a mean hue. The mean hues for target and possible distractor ensembles were selected with different criteria in Experiments 1 and 2; the details for Experiment 1 are under the main Methods section and for Experiment 2 under its Methods subsection. The mean hue for comparison ensembles was varied in 11 levels of difference from target ensemble mean hue.

### Procedure

At the start of the experiment, observers performed a simple color categorization task. A colored square of 3° in visual angle was shown in the middle of the screen, and the observer chose a color category that best matched the stimulus from the common 11 color categories (Jameson & Webster, 2019), excluding black, white, and gray. Stimulus colors included 72 values of equal steps around the hue circle. Each hue was repeated four times, and trials were presented in random order.

**Figure 3.**
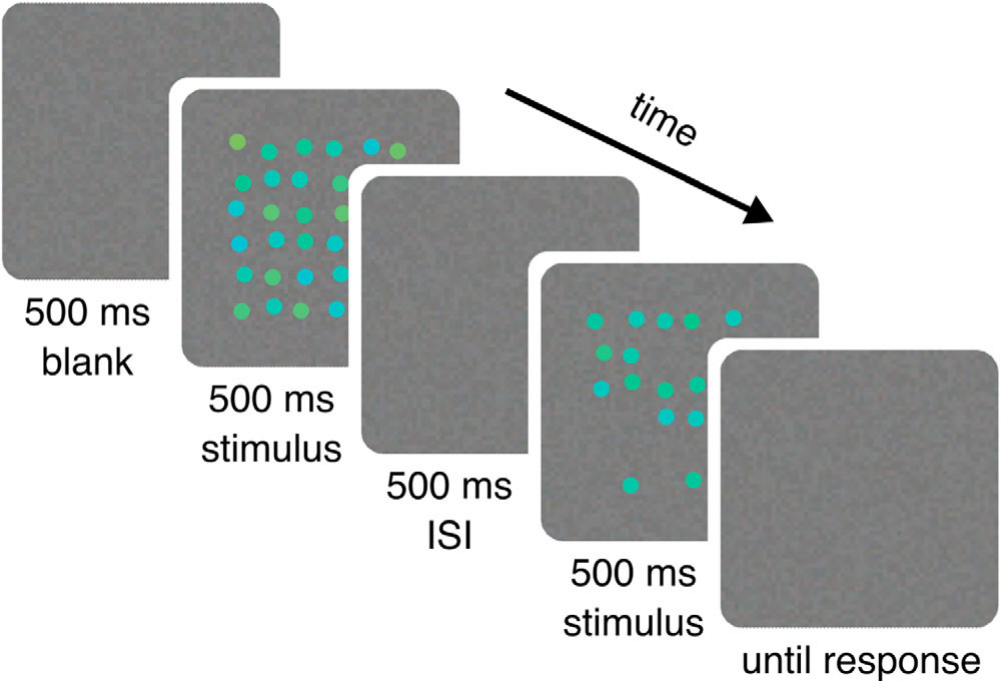
Time course of one trial. A blank screen was followed by the presentation of the first stimulus, an ISI, and the second stimulus, each lasting 500 ms. Finally, there was no stimulus (besides chromatic noise) on the screen until the observer gave their response.

In the ensemble discrimination task, each trial began with a blank screen presented for 500 ms. The first stimulus was then shown for 500 ms, followed by a 500-ms blank interstimulus interval (ISI) and the second stimulus for 500 ms (Figure 3). One of the two stimuli was always the test (target and possible distractor) and the other was the comparison, with a randomized order of presentation. The observer’s task was to compare the average hue of the target and comparison ensembles while ignoring the distractor ensemble, and to respond whether the ensemble in the latter interval was “yellower/greener” or “bluer/purpler” than in the first interval.

**Figure 4.**
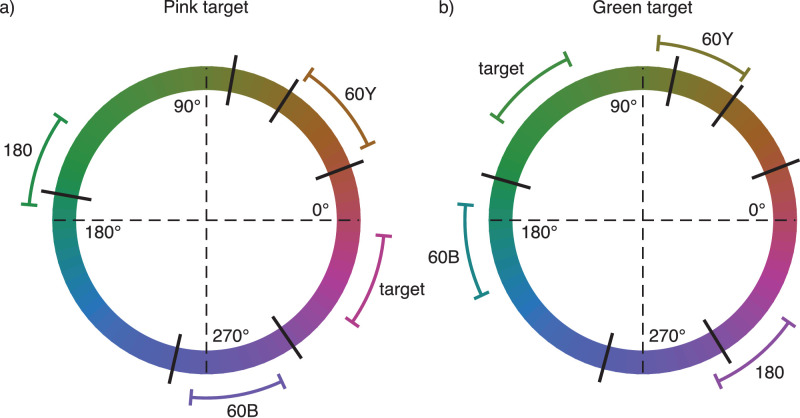
Average stimulus ensemble hue distributions in Experiment 1 displayed against a hue circle in CIELUV color space (a) for the pink target and (b) for the green target. Black lines orthogonal to the hue circle show average color category boundaries across observers. Curves tangential to the hue circle show the range of different ensemble hue distributions, not including trial-to-trial jitter.

In Experiment 1, observers were divided into two groups; the pink group had their target ensemble mean hue angle at 340° and the green group had their target ensemble mean hue angle at 130°. These were chosen to lie approximately in the middle of the pink and green color categories. In both cases, there were four experimental conditions: (1) no distractor ensemble or “baseline,” (2) distractor ensemble mean from the opposite side of the hue circle or “180,” (3) distractor ensemble mean 60° in hue angle from target ensemble mean toward yellow or “60Y,” and (4) distractor ensemble mean 60° in hue angle from target ensemble mean toward blue or “60B” (see Figure 4 for illustration). The motivation for the two 60° distractor conditions was to enable us to separate effects of the distractors from biases related to inhomogeneities of the color space or observers’ strategies in the task.

All ensembles had their dot hues drawn from a uniform distribution with 30° range along the hue circle. The target and distractor mean hues were varied by a common jitter value between ±7.5° in hue angle, randomized for each trial. The comparison ensemble mean hue was varied around target ensemble mean hue in 11 equal steps. One of the values was equal to target ensemble mean hue, with five steps in both positive and negative hue angle. The step sizes in hue angle for different experimental conditions were 4° for baseline, 5° for 180, and 6° for both 60Y and 60B.

To help observers implicitly learn to distinguish the target from distractor distributions, observers completed short practice blocks with trial-by-trial feedback for each experimental condition before the main task. We assumed observers’ percepts might be biased when a distractor ensemble was present. Giving feedback based on ground truth could thus be in conflict with observers’ percepts and artificially bias their responses toward the ground truth. To minimize such an effect, comparison levels closest to target mean hue were excluded in the practice blocks, and only the three most distant comparison levels in both negative and positive hue angle were used. Each practice block had 18 trials, with the whole practice consisting of 72 trials.

In the main task, each of the four blocks was repeated twice, and the order of all blocks was randomized. In each block, there were 10 repetitions for each of the 11 comparison levels. Thus, the experiment consisted of 880 trials and took observers approximately 45 minutes to complete. Including the categorization task and practice, the whole experiment lasted approximately 1 hour and 15 minutes.

Observers were given written instructions at the beginning of each practice and main task block with example test and comparison stimuli. The experimenter also gave the same instructions verbally with examples. Practice began with the baseline condition and instructions to compare the average hues of the two trial ensembles. Next practice block was the 180-condition with the clearest distinction between target and distractor elements. Observers were told that one of the two stimuli now also had elements of a different hue that they were to try and ignore and only compare the two task-relevant ensembles. Targets and distractors were pointed out from the example stimuli. Observers were also told that throughout a trial block, the distractor ensemble would be similarly related to the target ensemble as in the example. The last two blocks in the practice were the remaining distractor conditions (60Y and 60B in Experiment 1) in random order.

### Data analysis

From the color categorization data, we estimated individual color category boundaries. To delete any false key presses in the data that might skew category boundary estimation, two exclusion processes were used. First, if a color category had fewer than four responses overall, those responses were excluded, and the respective color category was not included in estimation. Second, if a response was separated from the main group of responses for that color category by 20° in hue angle or more, these isolated responses were excluded. A group of responses was defined as adjacent stimulus hue values with the same category response, and the cutoffs for the group were determined by three consecutive steps without that category response. The main group was the largest identified group in terms of number of hue steps. Finally, color category centroids were calculated as a circular mean across all responses for each category.

To estimate the color category boundaries, we defined a model observer that makes a category judgment based on a noisy hue measurement and stable category boundaries on the hue circle. The probability of a measurement given a stimulus was described by a von Mises probability density centered on the stimulus hue angle. Category boundaries were defined as points on the hue circle. When fitting the model, the free parameters were the von Mises concentration parameter κ and the category boundaries, each constrained to lie between two successive category centroids (determined individually for each observer, see above). For a given stimulus value, probabilities for category responses were calculated by integrating the area under the von Mises distribution between the boundary values of each category. This was done for each stimulus value to produce a matrix of category response probabilities for all stimuli and all categories. Optimal values for the free parameters were found by minimizing the negative log-likelihood of the parameters given the response counts of the observer.

For data from the ensemble mean comparison task, cumulative Gaussian psychometric functions (PMFs) were fit to the proportion of responses for the choice in the direction of a higher hue angle. For example, from the response options of “yellower” or “bluer,” the former is in the direction of a higher hue angle for the pink target and the latter for the green target (counterclockwise in Figure 4). The best-fitting mean and standard deviation (*SD*) parameters were found using a maximum likelihood method. For two observers, the PMF curve did not reach 0.25 or 0.75 probability within the comparison stimulus range in one experiment block. These two fits were unlikely to provide reliable estimates, and they were excluded from further analysis. The difference between PMF mean and ground truth was used as a measure of perceptual bias, and the standard deviation of the PMF was used as a measure of the discrimination threshold.

Comparisons between experimental conditions were performed for both bias and discrimination threshold measures. To enable meaningful comparisons, bias sign was set so that a positive value always indicated bias toward the distractor distribution hue (if applicable). Initial analysis was done using linear mixed models with experimental condition as an explanatory variable within observers and color group as an explanatory variable between observers. Groups were then compared pairwise using paired sample *t*-tests. Finally, effects of color category boundaries and discrimination thresholds on observers’ biases were tested by calculating Pearson correlation coefficients across observers within each condition. Two-way correlations between bias, discrimination threshold, and the two nearest color category boundaries to target were included. All *p*-values from tests with multiple comparisons were Bonferroni-corrected.

## Color categorization

**Figure 5.**
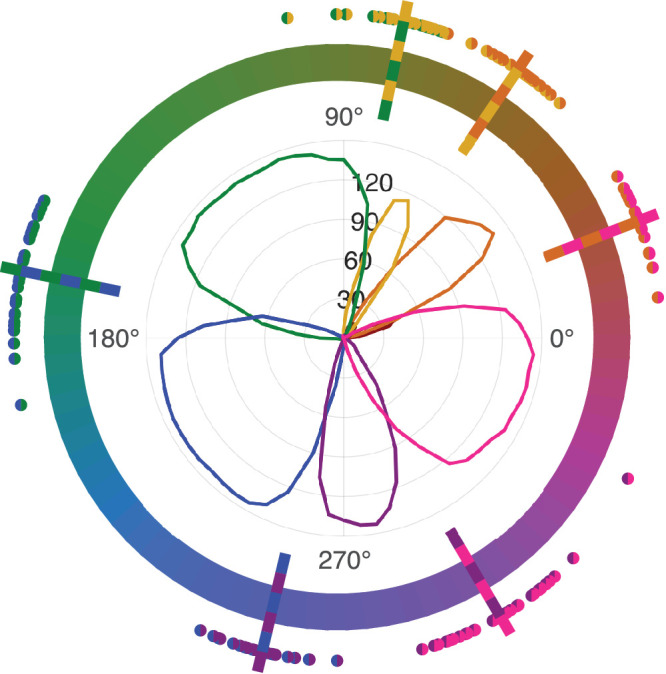
Color categorization results and estimated color category boundaries for observers, displayed against a hue circle in CIELUV color space. The graph inside the hue circle shows the counts across observers of assigning the corresponding hue on the hue circle into the color category corresponding to line color. The dots around the hue circle show estimated color category boundaries for observers and lines their respective means across observers. The two colors indicate the color categories separated by the boundary.

Other than the color categories red and brown, hues were assigned into categories fairly consistently both within and across observers. Only a subset of observers used red and brown in their responses at all. Thus, the representation of red and brown in the average data is small. Plotted against stimulus hues located on the hue circle, Figure 5 illustrates the sum of color category responses for all observers. Estimated color category boundaries exhibited some individual variation, summarized in Table 1. Average color category boundaries with their respective range of individual values are superimposed on Figure 5, excluding the red and brown categories.

**Table 1.** Minimum, mean, and maximum values of estimated color category boundaries across all observers, in degrees of hue angle in CIELUV cylindrical coordinates.

Boundary	Min	Mean	Max
orange-yellow	46.5	56	70
yellow-green	70.9	78.1	98.8
green-blue	155	168.1	192.1
blue-purple	245	256.6	270
purple-pink	285.3	300.3	334.1
pink-orange	7.1	21.1	29.9
red-orange	19.8	26.2	31.3
pink-red	4	10.2	16.2
orange-brown	37.3	46.7	61.1
brown-yellow	46.7	54.9	59.7

## Experiment 1

In Experiment 1, we investigated if the visual system can segment two hue distributions based only on hue and selectively form an ensemble percept for just one of the two distributions. Additionally, we examined how the distance and direction of the two distributions on the hue circle affects ensemble percepts of the target distribution.

### Results

**Figure 6.**
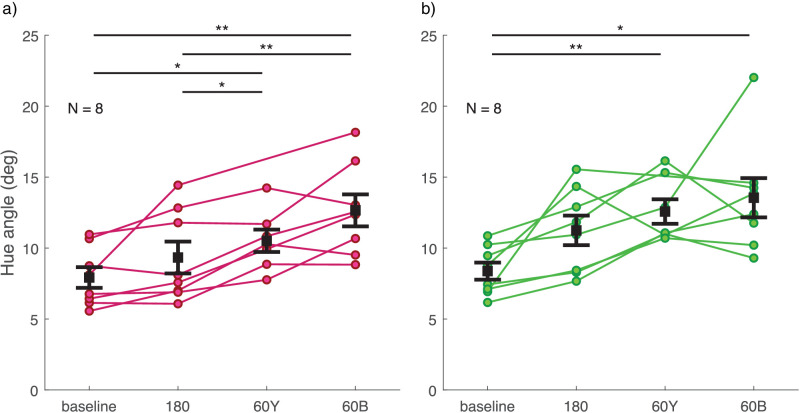
Discrimination thresholds for the average hue of target and comparison ensemble stimuli in Experiment 1 (a) for the pink group and (b) for the green group. Different experimental conditions are plotted on the x-axis and PMF *SD* is plotted on the y-axis as a measure of discrimination threshold. Each set of dots with connecting lines represents one observer. The error bar shows ± 1 *SEM* around the group mean. *p < 0.05. **p < 0.01.

Discrimination thresholds for all observers are illustrated in Figure 6, separately for the two color groups. There was no significant difference in thresholds between the two color groups (main effect or interaction with experimental condition). Compared to baseline, discrimination was significantly poorer in the 60Y- and 60B-conditions (pink group, 60Y: *t*(6) = −4.788, *p* = 0.018, 60B: *t*(7) = −5.489, *p* = 0.006; green group, 60Y: *t*(6) = −6.925, *p* = 0.003, 60B: *t*(7) = −4.388, *p* = 0.019). Additionally, in the pink group, discrimination was significantly poorer with the 60Y and 60B distractor distributions compared to the 180 distractor distribution condition (60Y: *t*(6) = −4.152, *p* = 0.036, 60B: *t*(7) = −6.305, *p* = 0.002).

**Figure 7.**
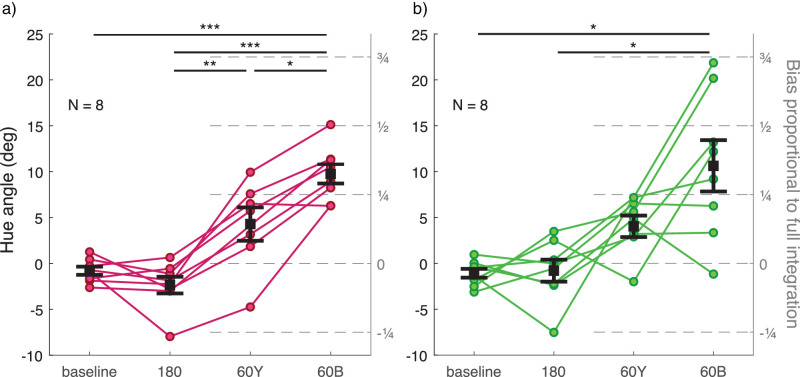
Bias in the perceived hue of the target ensemble stimulus in Experiment 1 (a) for the pink group and (b) for the green group. Different experimental conditions are plotted on the x-axis and PMF mean is plotted on the left-hand side y-axis as a measure of perceptual bias. Positive values on the y-axis for conditions 60Y and 60B always indicate bias toward distractor hues. Right-hand side y-axis shows bias magnitude as a proportion of expected bias if observers averaged all test stimulus elements (both target and distractor). Each set of dots with connecting lines represents one observer. The error bar shows ± 1 *SEM* around group mean. *p < 0.05. **p < 0.01. ***p < 0.001.

Perceptual bias data for all observers are illustrated in Figure 7, separately for the two color groups. There was no significant difference in biases between the two color groups (main effect or interaction with experimental condition). Overall, biases tended to be larger in conditions with distractors close to the target distribution in hue. In the 60Y-condition, the bias was on average a little less and, in the 60B-condition, a little more than one fourth of what would be expected if observers averaged all elements in the test stimulus (see right-hand side y-axis in Figure 7). Bias toward the distractors in the 60B-condition was statistically different from baseline and from the 180-condition in both color groups (pink group, vs. baseline: *t*(7) = −8.053, *p* < 0.001, vs. 180: *t*(7) = −9.922, *p* < 0.001; green group, vs. baseline: *t*(7) = −3.938, *p* = 0.034, vs. 180: *t*(7) = −3.732, *p* = 0.044). However, the bias in the 60Y-condition was significantly different from 180 only in the pink group (vs. 180: *t*(6) = −5.770, *p* = 0.007, vs. 60B: *t*(6) = −4.133, *p* = 0.037). As expected, bias for distractors 180 degrees away from the target did not differ from baseline in either color group.

As is clear from Figures 6 and 7, there were substantial individual differences in the pattern of discrimination thresholds and bias across distractor conditions, especially in the green group. We asked whether this might be due to individual variation in the two color category boundaries around the target stimulus distribution, visible in Figure 5. Correlation analyses between bias, discrimination threshold, and color category boundaries did not support this: The only significant correlation was found between discrimination threshold and bias in the 60Y-condition of both color groups (pink group: Pearson’s *r* = 0.967, *p* < 0.001; green group: Pearson’s *r* = 0.928, *p* = 0.010). All other correlations were nonsignificant (all *p* > 0.639).

## Experiment 2

In Experiment 1, we found that observers were able to form hue ensemble percepts for targets among distractors, although distractor similarity affected both precision and bias. Measuring color categories in Experiment 1 was aimed at verifying that our target hue distributions were within the pink and green categories and to probe for effects of individual differences. In Experiment 2, we set out to investigate whether color categories affect the segmentation of ensemble distributions and how they affect the perceived average of the target distribution. Specifically, we examined if ensemble percepts differ depending on whether the target and distractor distributions were within the same color category or in different color categories. Experiment 2 used the same methods as Experiment 1 except for the differences detailed below.

### Methods

In Experiment 2, after completing the color categorization task, observers were presented with a second categorization task to more accurately estimate their green-blue boundary. Observers had only two choices for responses: green or blue. The stimulus was otherwise identical to the one in the previous color categorization task, but hue angle was varied in four interleaved staircases. Two staircases started with −20° in hue angle and two staircases with +20° in hue angle from the green-blue category boundary estimated from the first color categorization task. Responding “green” increased hue angle, and responding “blue” decreased hue angle. Step size started at 4° in hue angle and was decreased by one fourth for each reversal in the staircase. Each staircase continued for 25 trials, and the green-blue boundary was calculated as the mean of the hue angle values of the last two reversal points of all four staircases.

**Figure 8.**
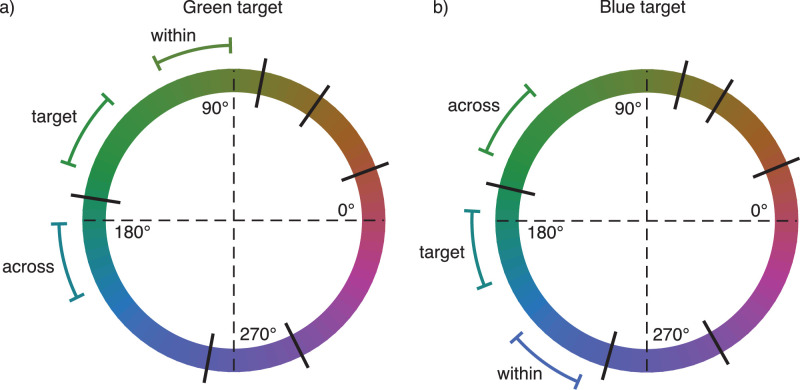
Average stimulus ensemble hue distributions in Experiment 2 displayed against a hue circle in CIELUV color space (a) for the green target and (b) for the blue target. Black lines orthogonal to the hue circle show average color category boundaries across observers. Curves tangential to the hue circle show the range of different ensemble hue distributions, not including trial-to-trial jitter.

For the ensemble discrimination task, observers were again divided into two groups. The green group had their target ensemble mean hue angle at −22.5° from their green-blue boundary, and the blue group had their target ensemble mean hue angle at +22.5° from their green-blue boundary. There were three experimental conditions: (1) “baseline”: no distractor ensemble, (2) “across”: distractor ensemble mean across the green-blue boundary (+45° in hue angle from target ensemble mean hue for green group, −45° for blue group), and (3) “within”: distractor ensemble mean within the target ensemble color category (−45° from target ensemble mean hue for green group, +45° for blue group). Figure 8 illustrates the experimental conditions.

To be able to fit both target and distractor distributions within the same color category, Experiment 2 employed slightly smaller variation in distributions than Experiment 1. The uniform distribution, from which ensemble dot hues were drawn, had 25° range along the hue circle. Additionally, target and distractor mean hues were randomized by a common jitter value between ± 5° in hue angle for each trial. The comparison ensemble mean hue was varied around target ensemble mean hue in 11 equal steps, with one equal to zero and five steps in both positive and negative hue angle. The step sizes in hue angle for different experimental conditions were 4 degrees for baseline, and 7 degrees for both across- and within-conditions.

Each of the 11 comparison levels had eight repetitions in one block, and each block was repeated three times. The order of all blocks was randomized. In total, the main discrimination task had 792 trials and took observers approximately 40 minutes. Note that in addition to the three experimental conditions in Experiment 2, practice included a 180° distractor block as in Experiment 1 to ease observers into the task with distractors. To further aid observers in identifying target and distractor elements, each trial block was preceded by 11 dummy trials during which the distractor ensemble dots grew trial-by-trial from 0° to their final size of 0.5° in equal increments. With the two categorization tasks and practice, the whole experiment lasted approximately 1 hour and 20 minutes.

### Results

**Figure 9.**
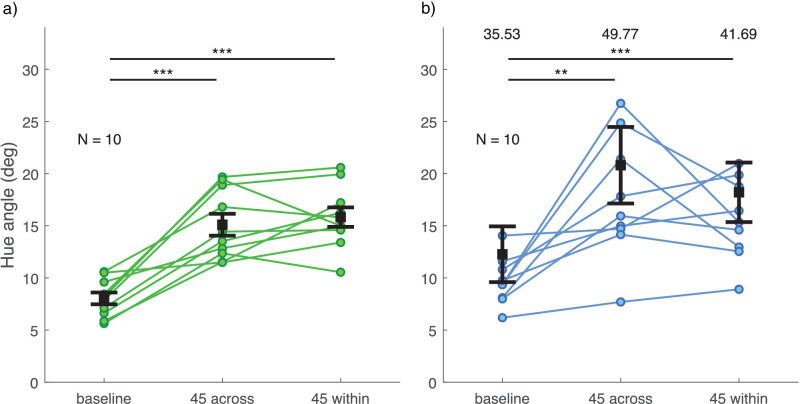
Discrimination thresholds for the average hue of target and comparison ensemble stimuli in Experiment 2 (a) for the green group and (b) for the blue group. Different experimental conditions are plotted on the x-axis and PMF *SD* is plotted on the y-axis as a measure of discrimination threshold. The green group and the blue group are plotted in separate graphs in their respective colors. Each set of dots with connecting lines represents one observer. The error bar shows ± 1 *SEM* around group mean. **p < 0.01. ***p < 0.001.

Discrimination thresholds for all observers are illustrated in Figure 9, separately for the two color groups. There was no significant difference in thresholds between the two color groups (main effect or interaction with experimental condition). Compared to baseline, discrimination was poorer with a distractor distribution (green group, across: *t*(9) = −6.516, *p* < 0.001, within: *t*(9) = −7.297, *p* < 0.001; blue group, across: *t*(9) = −4.393, *p* = 0.005, within: *t*(9) = −8.312, *p* < 0.001). There was no significant difference in discrimination between the within- and across-conditions. However, observers varied significantly in the pattern of precision across the two distractor conditions. Most notably, three observers in the blue group had a clearly higher threshold in the across-condition compared to the within-condition. The opposite pattern could be seen in one observer in both groups.

**Figure 10.**
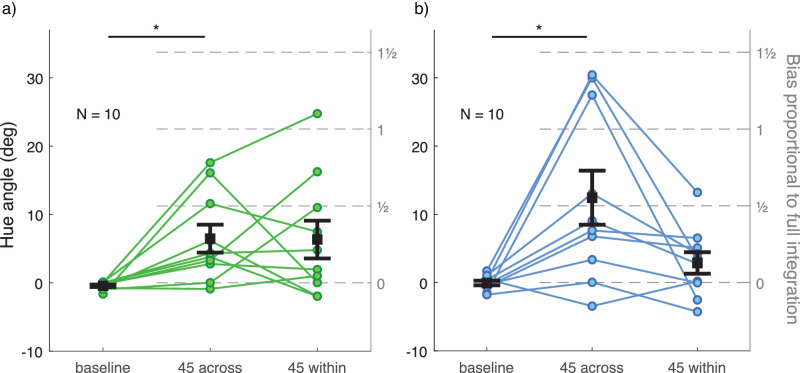
Bias in the perceived hue of the target ensemble stimulus in Experiment 2 (a) for the green group and (b) for the blue group. Different experimental conditions are plotted on the x-axis and PMF mean is plotted on the left-side y-axis as a measure of perceptual bias. Positive values on the y-axis for conditions across and within always indicate bias toward distractor hues. Right-hand side y-axis shows bias magnitude as a proportion of expected bias if observers averaged all test stimulus elements (both target and distractor). Each set of dots with connecting lines represents one observer. The error bar shows ± 1 *SEM* around group mean. *p < 0.05.

Perceptual biases are illustrated in Figure 10, separately for the two color groups. There was no significant difference in biases between the two color groups (main effect or interaction with experimental condition). In the baseline condition with no distractor distribution, hue percepts were unbiased. With a distractor distribution in the stimulus, hue percepts were mostly biased toward distractor hues. In three of the four distractor conditions, the average bias was roughly one fourth of what would be expected if observers averaged all elements in the test stimulus; in the across-condition of the blue group, average bias was approximately one half instead of one fourth (see right-hand side y-axis in Figure 10). The average bias was significantly different from baseline only in the across-condition for both color groups (green group: *t*(9) = −3.364, *p* = 0.025, blue group: *t*(9) = −3.212, *p* = 0.032). The amount and pattern of bias depended strongly on the observer. In most cases, a strong bias was only present in either the across- or within-condition for any one observer. In the green group, one observer had a strong bias only in the across-condition and two observers only in the within-condition. In the blue group, three observers had a strong bias only in the across-condition. In one case in the green group (within-condition) and in three cases in the blue group (across-condition), the proportion of bias compared to full integration was over 1. This means that in these conditions, observers weighted the distractor distribution *more* than the target distribution. Notably, observer biases were fairly consistent through all three repetitions of a block if PMFs were fit separately for each repetition (range between lowest and highest was 5.07° in hue angle on average). If observers were mistaken about their task, they were so consistently and regardless of the instructions and dummy trials repeated at the beginning of each block repetition.

Similarly to Experiment 1, individual variation in the two color category boundaries around the target stimulus distribution could not explain variation in any of the discrimination threshold or bias measures (all *p* > 0.346). There was no significant correlation between discrimination threshold and bias in any experimental condition (all *p* > 0.073).

## Discussion

We investigated whether the visual system can selectively form an ensemble percept from one of two spatially intermixed hue distributions, and how this depends on target-distractor difference and category boundaries. We used a novel approach where the observers had to segment the appropriate ensemble elements based on hue distribution alone while also estimating their average hue. We found that observers were able to form an accurate and precise target ensemble percept in the presence of distractors sampled from the opposite side of the hue circle. However, ensemble percepts were biased and less precise with distractor hues closer to the target hues. Finally, we found no overall effect of category boundaries on precision or bias.

To form an ensemble percept of target elements among distractors, the visual system needs to segment relevant color information. According to subsampling accounts of ensemble perception (see, e.g., Marchant, Simons, & de Fockert, 2013), a subset of elements is chosen by focused attention, while other accounts suggest that focused attention filters relevant information from a population response (e.g., Baek & Chong, 2020). In either case, inaccurate segmentation leads to the inclusion of distractor elements or responses in the target mean estimate. Such failures of segmentation should both increase uncertainty as well as mix distractor signals in the ensemble estimate. We saw this trend in all distractor conditions where the distractor distribution mean was 45° or 60° away from the target mean. Although the bias was always in the direction of the distractors, its magnitude was on average only 25% of that of full integration, showing that observers were able to separate the targets to a large extent. In contrast, even if observers were integrating the 180° distractors in the estimate, there should be no bias on average, because there is no meaningful prediction for the average of opposing hues. Nevertheless, a mixed representation would still be considerably noisier than one without distractors due to the large variation in hue, leading to higher discrimination thresholds. There was no bias in the 180° distractor condition and only a small, not statistically significant elevation in the average discrimination thresholds compared to baseline. This indicates little mixing of target and distractor distributions and suggests accurate segmentation in this condition.

Experiment 2 showed that category membership does not facilitate ensemble segmentation: Observers were able to segment targets with equal accuracy and precision for within-category and across-category distractors. If anything, segmentation tended to be more accurate for within-category distractors, going against the categorical facilitation hypothesis. Categorical facilitation across the green-blue boundary has been found in many previous studies using one-item stimuli (Drivonikou et al., 2007; Franklin, Drivonikou, Bevis, et al., 2008; Franklin, Drivonikou, Clifford, et al., 2008; Gilbert, Regier, Kay, & Ivry, 2006; Witzel & Gegenfurtner, 2011, but see Witzel & Gegenfurtner, 2015), but our results are in line with previous studies investigating color ensemble perception specifically. The number of different color categories a hue ensemble includes does not influence averaging performance for the whole set (Maule & Franklin, 2015). Webster, Kay, and Webster (2014) also found that matching the average of a two-color hue ensemble to an internal criterion showed little categorical bias in weighting of the hue components. Furthermore, in a task where observers were asked if a probed hue had been a member of a seen ensemble, Maule, Witzel, and Franklin (2014) found that observers reported hues close to a category boundary as a member more often than hues close to the category prototype. Maule, Witzel, and Franklin (2014) speculate that individual elements near color category boundaries might have a more unstable categorical identity and are thus harder to identify as not being a member in the ensemble. This idea is supported by findings of categorical facilitation for one-item stimuli *within* a category, showing improved discrimination (Hanley & Roberson, 2011) and lower error rates and response times (Wright, 2012) when the target was a more typical exemplar of the category color. Interestingly, in Experiment 2, 4 observers out of 20 showed a considerably stronger bias in the across-condition compared to the within-condition (a difference of more than half of full integration as the criterion). This trend is consistent with less accurate grouping for hues straddling a category boundary (see Maule, Witzel, & Franklin, 2014), leading to more bias through poorer segmentation.

While we did not find a difference related to category membership in Experiment 2, in Experiment 1, bias between the two 60° conditions depended on the direction of the distractors relative to the targets. Interestingly, the 60Y- and 60B-condition biases were roughly comparable for the green and pink groups, despite the colors being sampled from different regions of the hue circle. The possible larger-scale inhomogeneity in our choice of colors is thus worth some consideration. Equal distance in nominally uniform color spaces such as CIELUV is aimed to produce equal discriminability, but only within a small range of similar hues, and only for the average observer (see, e.g., Brainard, 2003). In Experiment 1, we found a stronger bias for bluer compared to yellower distractors despite both being nominally equidistant in hue from the target distribution. This is broadly in line with known perceptual asymmetries on the blue-yellow axis: Achromatic settings tend to cluster toward bluish from the adapting chromaticity (e.g., Chauhan et al., 2014), and illumination discrimination is poorer for changes toward blue compared to yellow (Aston, Radonjić, Brainard, & Hurlbert, 2019; Pearce, Crichton, Mackiewicz, Finlayson, & Hurlbert, 2014). Another way to think of the yellow/blue asymmetry is in terms of the distinction between warm and cool colors. Objects tend to exhibit warmer colors than backgrounds (Gibson et al., 2017; Rosenthal et al., 2018), and it is argued that pressure for more accurate color-naming efficiency with warm colors is reflected in how linguistic color categories develop (Conway, Ratnasingam, Jara-Ettinger, Futrell, & Gibson, 2020; Gibson et al., 2017). Colors straddling the warm-cool boundary can exhibit categorical facilitation irrespective of basic color categories (Holmes & Regier, 2017). The higher discriminability of the yellowish/warm distractors could make it easier to ignore them in the target ensemble estimate, leading to the observed asymmetry in bias for the bluish and yellowish distractors in Experiment 1, while the green-blue boundary in Experiment 2 may not have produced a large enough distinction to affect biases. Our present data, however, do not offer a strong test of these hypotheses.

Other stimulus-related aspects might have exerted some influence on our results. First, as our stimuli were within a limited hue range, observer responses were susceptible to central tendency bias (CTB), a bias toward the grand mean across trials (Hollingworth, 1910). In both experiments, across all blocks, this grand mean would equal the target mean, and CTB would effectively reduce the bias toward distractors. Could observers have in fact integrated both target and distractor ensembles if we take CTB into account? The expected bias from fully integrating both ensembles is seen on the right-hand side y-axis of Figures 7 and 10. To account for the average results, observers would have had to weight the grand mean (CTB) *more* than the sensory response in a given trial. We find this unlikely because CTB in hue estimation is generally weaker in magnitude and not found at all with a short ISI such as we used here (Olkkonen, McCarthy, & Allred, 2014).

A second potentially confounding factor is the configural differences between the target-only (baseline) and target-plus-distractor ensembles. Specifically, target-plus-distractor ensembles were denser with more elements and had a more regular spatial structure. These features could lead to poorer ensemble segmentation through crowding and stronger grouping, respectively. The fact that there was no significant difference in discrimination thresholds between the baseline and 180-conditions in Experiment 1 suggests, however, that spatial crowding and grouping did not play a significant role in the present study. Instead, similarity of target and distractor colors may itself increase crowding effects (Kennedy & Whitaker, 2010). Color similarity is also found to add to the spatial grouping cues (Baylis & Driver, 1992; Farmer & Taylor, 1980), which may in turn influence the color percept (Corbett, 2017; van Lier & Wagemans, 1997). Thus, configural factors may play some role in conditions other than the 180-condition, but to fully rule out these effects would require further study.

The amount of bias varied substantially across individual observers in some experimental conditions. This variability was not explained by individual variation in color category boundaries or discrimination thresholds. Qualitatively comparing Experiments 1 and 2 may offer insight into potential observer strategies influencing the results. In Experiment 1, where the difference between target and distractor hues was on average larger than in Experiment 2, perceptual biases were smaller and more consistent across observers. In Experiment 2 with the smaller difference between targets and distractors, perceptual biases varied considerably between observers. Discrimination thresholds were overall higher in Experiment 2, indicating that the segmentation task was more difficult presumably due to the smaller hue difference between targets and distractors. We speculate that this difficulty might lead some observers to be more aware that they might accidentally mix in distractor elements in their estimates and to compensate for their estimated visual bias in their responses. This could then lead to a smaller bias than for observers not employing such strategies and thus to more variation between observers. In conjunction, cognitive compensation for assumed bias in responses would break the straightforward correlation between precision and bias predicted by simple mixing of target and distractor signals. Lastly, if observers were to rely on such strategies only in specific experimental conditions, it could lead to the dramatic within-observer differences seen in Experiment 2 biases (Figure 10).

Multiple recent studies have found that ensemble statistics are already represented in early visual areas alongside individual feature representations, even when they are unrelated to the behavioral task (Epstein & Emmanouil, 2021; Jia, Wang, Chen, Ding, & Fang, 2022; Tark, Kang, Chong, & Shim, 2021; Zhao et al., 2023). The lack of categorical facilitation in our results may indicate that segmentation in hue ensemble perception can also be achieved by early visual processing based mainly on sensory information. Behavioral studies suggest that categorical facilitation is induced when task demands draw attention to color labels but not when tasks are performed by purely perceptual decisions (Webster & Kay, 2012; Witzel & Gegenfurtner, 2015). The same may apply for segmentation in hue ensemble perception.

## Conclusions

We showed that observers were able to form hue ensemble percepts selectively over one of two spatially intermixed hue distributions. This provides evidence that the visual system can make use of color ensembles even in environments with a variety of differently colored objects and aid in parsing the visual scene. Percepts were generally biased toward task-unrelated hues, and difference in hue between distributions was the major determinant for the precision and bias of the ensemble percept, while categorical identity had no effect. Lack of a straightforward relationship between discrimination thresholds and bias indicates that inaccurate stimulus segmentation is not the only factor explaining target ensemble percepts.

## References

[bib1] Alvarez, G. A. (2011). Representing multiple objects as an ensemble enhances visual cognition. *Trends in Cognitive Sciences,* 15(3), 122–131, 10.1016/j.tics.2011.01.003. 21292539

[bib2] Ariely, D. (2001). Seeing sets: Representation by statistical properties. *Psychological Science,* 12(2), 157–162, 10.1111/1467-9280.00327. 11340926

[bib3] Aston, S., Radonjić, A., Brainard, D. H., & Hurlbert, A. C. (2019). Illumination discrimination for chromatically biased illuminations: Implications for color constancy. *Journal of Vision,* 19(3), 15, 10.1167/19.3.15. PMC6440550 30924843

[bib4] Bae, G. Y., Olkkonen, M., Allred, S. R., & Flombaum, J. I. (2015). Why some colors appear more memorable than others: A model combining categories and particulars in color working memory. *Journal of Experimental Psychology: General,* 144(4), 744, 10.1037/xge0000076. 25985259

[bib5] Baek, J., & Chong, S. C. (2020). Ensemble perception and focused attention: Two different modes of visual processing to cope with limited capacity. *Psychonomic Bulletin & Review,* 27, 602–606, 10.3758/s13423-020-01718-7. 32128720

[bib6] Baylis, G. C., & Driver, J. (1992). Visual parsing and response competition: The effect of grouping factors. *Perception & Psychophysics,* 51(2), 145–162, 10.3758/BF03212239. 1549433

[bib7] Brainard, D. H. (1997). The psychophysics toolbox. *Spatial Vision,* 10, 433–436, 10.1163/156856897X00357. 9176952

[bib8] Brainard, D. H. (2003). Color appearance and color difference specification. In S. K. Shevell (Ed.), *The science of color* (2nd ed., pp. 191–216). Oxford, UK: Elsevier Science.

[bib9] Chauhan, T., Perales, E., Xiao, K., Hird, E., Karatzas, D., & Wuerger, S. (2014). The achromatic locus: Effect of navigation direction in color space. *Journal of Vision,* 14(1), 25, 10.1167/14.1.25. PMC3903293 24464164

[bib10] Chetverikov, A., Campana, G., & Kristjánsson, Á. (2017). Representing color ensembles. *Psychological Science,* 28(10), 1510–1517, 10.1177/0956797617713787. 28862923

[bib11] Chetverikov, A., Campana, G., & Kristjánsson, Á. (2020). Probabilistic rejection templates in visual working memory. *Cognition,* 196, 104075, 10.1016/j.cognition.2019.104075. 31841813

[bib12] Chong, S. C., & Treisman, A. (2003). Representation of statistical properties. *Vision Research,* 43(4), 393–404, 10.1016/S0042-6989(02)00596-5. 12535996

[bib13] Conway, B. R., Ratnasingam, S., Jara-Ettinger, J., Futrell, R., & Gibson, E. (2020). Communication efficiency of color naming across languages provides a new framework for the evolution of color terms. *Cognition,* 195, 104086, 10.1016/j.cognition.2019.104086. 31731116 PMC6939132

[bib14] Corbett, J. E. (2017). The whole warps the sum of its parts: Gestalt-defined-group mean size biases memory for individual objects. *Psychological Science* 28(1), 12–22, 10.1177/0956797616671524. 27879322

[bib15] D'Zmura, M. (1991). Color in visual search. *Vision Research,* 31(6), 951–966, 10.1016/0042-6989(91)90203-H. 1858326

[bib16] Dakin, S. C., & Watt, R. J. (1997). The computation of orientation statistics from visual texture. *Vision Research,* 37(22), 3181–3192, 10.1016/S0042-6989(97)00133-8. 9463699

[bib17] De Gardelle, V., & Summerfield, C. (2011). Robust averaging during perceptual judgment. *Proceedings of the National Academy of Sciences,* 108(32), 13341–13346, 10.1073/pnas.1104517108. PMC3156162 21788517

[bib18] Drivonikou, G. V., Kay, P., Regier, T., Ivry, R. B., Gilbert, A. L., Franklin, A., … Davies, I. R. (2007). Further evidence that whorfian effects are stronger in the right visual field than the left. *Proceedings of the National Academy of Sciences,* 104(3), 1097–1102, 10.1073/pnas.0610132104. PMC1783370 17213312

[bib19] Ennis, R., Schiller, F., Toscani, M., & Gegenfurtner, K. R. (2018). Hyperspectral database of fruits and vegetables. *Journal of the Optical Society of America A,* 35(4), B256–B266, 10.1364/JOSAA.35.00B256. 29603941

[bib20] Epstein, M. L., & Emmanouil, T. A. (2021). Ensemble statistics can be available before individual item properties: Electroencephalography evidence using the oddball paradigm. *Journal of Cognitive Neuroscience,* 33(6), 1056–1068, 10.1162/jocn_a_01704. 34428790 PMC8385227

[bib21] Farmer, E. W., & Taylor, R. M. (1980). Visual search through color displays: Effects of targetbackground similarity and background uniformity. *Perception & Psychophysics,* 27(3), 267–272, 10.3758/BF03204265. 7383809

[bib22] Franklin, A., Drivonikou, G. V., Bevis, L., Davies, I. R., Kay, P., & Regier, T. (2008). Categorical perception of color is lateralized to the right hemisphere in infants, but to the left hemisphere in adults. *Proceedings of the National Academy of Sciences,* 105(9), 3221–3225, 10.1073/pnas.0712286105. PMC2265127 18316729

[bib23] Franklin, A., Drivonikou, G. V., Clifford, A., Kay, P., Regier, T., & Davies, I. R. (2008). Lateralization of categorical perception of color changes with color term acquisition. *Proceedings of the National Academy of Sciences,* 105(47), 18221–18225, 10.1073/pnas.0809952105. PMC2587569 19015521

[bib24] Gegenfurtner, K. R., & Rieger, J. (2000). Sensory and cognitive contributions of color to the recognition of natural scenes. *Current Biology,* 10(13), 805–808, 10.1016/S0960-9822(00)00563-7. 10898985

[bib25] Gibson, E., Futrell, R., Jara-Ettinger, J., Mahowald, K., Bergen, L., Ratnasingam, S., … Conway, B. R. (2017). Color naming across languages reflects color use. *Proceedings of the National Academy of Sciences,* 114(40), 10785–10790, 10.1073/pnas.1619666114. PMC5635863 28923921

[bib26] Gilbert, A. L., Regier, T., Kay, P., & Ivry, R. B. (2006). Whorf hypothesis is supported in the right visual field but not the left. *Proceedings of the National Academy of Sciences,* 103(2), 489–494, 10.1073/pnas.0509868103. PMC1326182 16387848

[bib27] Goda, N., & Fujii, M. (2001). Sensitivity to modulation of color distribution in multicolored textures. *Vision Research,* 41(19), 2475–2485, 10.1016/S0042-6989(01)00136-5. 11483178

[bib28] Haberman, J., & Whitney, D. (2007). Rapid extraction of mean emotion and gender from sets of faces. *Current Biology,* 17(17), R751–R753, 10.1016/j.cub.2007.06.039. 17803921 PMC3849410

[bib29] Haberman, J., & Whitney, D. (2009). Seeing the mean: Ensemble coding for sets of faces. *Journal of Experimental Psychology: Human Perception and Performance,* 35(3), 718–734, 10.1037/a0013899. 19485687 PMC2696629

[bib30] Haberman, J., & Whitney, D. (2010). The visual system discounts emotional deviants when extracting average expression. *Attention, Perception, & Psychophysics,* 72(7), 1825–1838, 10.3758/APP.72.7.1825. PMC3123539 20952781

[bib31] Halberda, J., Sires, S. F., & Feigenson, L. (2006). Multiple spatially overlapping sets can be enumerated in parallel. *Psychological Science,* 17(7), 572–576, 10.1111/j.1467-9280.2006.01746.x. 16866741

[bib32] Hanley, J. R., & Roberson, D. (2011). Categorical perception effects reflect differences in typicality on within-category trials. *Psychonomic Bulletin & Review,* 18, 355–363, 10.3758/s13423-010-0043-z. 21327385

[bib33] Hansen, T., & Gegenfurtner, K. R. (2009). Independence of color and luminance edges in natural scenes. *Visual Neuroscience,* 26(1), 35–49, 10.1017/S0952523808080796. 19152717

[bib34] Hansmann-Roth, S., Kristjánsson, Á., Whitney, D., & Chetverikov, A. (2021). Dissociating implicit and explicit ensemble representations reveals the limits of visual perception and the richness of behavior. *Scientific Reports,* 11(1), 1–12, 10.1038/s41598-021-83358-y. 33594160 PMC7886863

[bib35] Hollingworth, H. L. (1910). The central tendency of judgment. *The Journal of Philosophy, Psychology and Scientific Methods,* 7(17), 461–469, 10.2307/2012819.

[bib36] Holmes, K. J., & Regier, T. (2017). Categorical perception beyond the basic level: The case of warm and cool colors. *Cognitive Science,* 41(4), 1135–1147. 27404377 10.1111/cogs.12393

[bib37] Iakovlev, A. U., & Utochkin, I. S. (2023). Ensemble averaging: What can we learn from skewed feature distributions? *Journal of Vision,* 23(1), 5, 10.1167/jov.23.1.5. PMC9832727 36602815

[bib38] Im, H. Y., & Chong, S. C. (2014). Mean size as a unit of visual working memory. *Perception,* 43(7), 663–676, 10.1068/p7719. 25223110

[bib39] Im, H. Y., Tiurina, N. A., & Utochkin, I. S. (2021). An explicit investigation of the roles that feature distributions play in rapid visual categorization. *Attention, Perception, & Psychophysics,* 83, 1050–1069, 10.3758/s13414-020-02046-7. 32410015

[bib40] Ishihara, S. (1987). *Test for colour-blindness**.* Tokyo, Japan: Kanehara.

[bib41] Jameson, K. A., & Webster, M. A. (2019). Color and culture: Innovations and insights since basic color terms—their universality and evolution (1969). *Color Research & Application,* 44(6), 1034–1041, 10.1002/col.22438.

[bib42] Jeong, J., & Chong, S. C. (2020). Adaptation to mean and variance: Interrelationships between mean and variance representations in orientation perception. *Vision Research,* 167, 46–53, 10.1016/j.visres.2020.01.002. 31954877

[bib43] Jia, J., Wang, T., Chen, S., Ding, N., & Fang, F. (2022). Ensemble size perception: Its neural signature and the role of global interaction over individual items. *Neuropsychologia,* 173, 108290, 10.1016/j.neuropsychologia.2022.108290. 35697088

[bib44] Juni, M. Z., Singh, M., & Maloney, L. T. (2010). Robust visual estimation as source separation. *Journal of Vision,* 10(14), 2, 10.1167/10.14.2. PMC4454090 21131562

[bib45] Kennedy, G. J., & Whitaker, D. (2010). The chromatic selectivity of visual crowding. *Journal of Vision,* 10(6), 15, 10.1167/10.6.15. 20884564

[bib46] Khayat, N., Fusi, S., & Hochstein, S. (2021). Perceiving ensemble statistics of novel image sets. *Attention, Perception, & Psychophysics,* 83, 1312–1328, 10.3758/s13414-020-02174-0. PMC8049939 33420715

[bib47] Khayat, N., & Hochstein, S. (2019). Relating categorization to set summary statistics perception. *Attention, Perception, & Psychophysics,* 81, 2850–2872, 10.3758/s13414-019-01792-7. PMC6856046 31243687

[bib48] Khvostov, V. A., Iakovlev, A. U., Wolfe, J. M., & Utochkin, I. S. (2024). What is the basis of ensemble subset selection? *Attention, Perception, & Psychophysics,* 86, 776–798, 10.3758/s13414-024-02850-5. 38351233

[bib49] Kim, M., & Chong, S. C. (2020). The visual system does not compute a single mean but summarizes a distribution. *Journal of Experimental Psychology: Human Perception and Performance,* 46(9), 1013, 10.1037/xhp0000804. 32496089

[bib50] Kingdom, F. A. (2003). Color brings relief to human vision. *Nature Neuroscience,* 6(6), 641–644, 10.1038/nn1060. 12740582

[bib51] Kleiner, M., Brainard, D., & Pelli, D. (2007). What's new in psychtoolbox-3? *Perception,* 36(ECVP Abstract Supplement), 14.

[bib52] Li, A., & Lennie, P. (1997). Mechanisms underlying segmentation of colored textures. *Vision Research,* 37(1), 83–97, 10.1016/S0042-6989(96)00152-6. 9068832

[bib53] Li, A., & Lennie, P. (2001). Importance of color in the segmentation of variegated surfaces. *Journal of the Optical Society of America A,* 18(6), 1240–1251, 10.1364/JOSAA.18.001240. 11393615

[bib54] Lindsey, D. T., & Brown, A. M. (2009). World color survey color naming reveals universal motifs and their within-language diversity. *Proceedings of the National Academy of Sciences,* 106(47), 19785–19790, 10.1073/pnas.0910981106. PMC2775038 19901327

[bib55] Lindsey, D. T., & Brown, A. M. (2021). Lexical color categories. *Annual Review of Vision Science,* 7, 605–631, 10.1146/annurev-vision-093019-112420. 34524876

[bib56] Marchant, A. P., Simons, D. J., & de Fockert, J. W. (2013). Ensemble representations: Effects of set size and item heterogeneity on average size perception. *Acta Psychologica,* 142(2), 245–250, 10.1016/j.actpsy.2012.11.002. 23376135

[bib57] Maule, J., & Franklin, A. (2015). Effects of ensemble complexity and perceptual similarity on rapid averaging of hue. *Journal of Vision,* 15(4), 6, 10.1167/15.4.6. 26114595

[bib58] Maule, J., & Franklin, A. (2016). Accurate rapid averaging of multihue ensembles is due to a limited capacity subsampling mechanism. *Journal of the Optical Society of America A,* 33(3), A22–A29, 10.1364/JOSAA.33.000A22. 26974927

[bib59] Maule, J., & Franklin, A. (2020). Adaptation to variance generalizes across visual domains. *Journal of Experimental Psychology: General,* 149(4), 662, 10.1037/xge0000678. 31464510

[bib60] Maule, J., Witzel, C., & Franklin, A. (2014). Getting the gist of multiple hues: Metric and categorical effects on ensemble perception of hue. *Journal of the Optical Society of America A,* 31(4), A93–A102, 10.1364/JOSAA.31.000A93. 24695209

[bib61] Norman, L. J., Heywood, C. A., & Kentridge, R. W. (2015). Direct encoding of orientation variance in the visual system. *Journal of Vision,* 15(4), 3, 10.1167/15.4.3. 26067349

[bib62] Olkkonen, M., McCarthy, P. F., & Allred, S. R. (2014). The central tendency bias in color perception: Effects of internal and external noise. *Journal of Vision,* 14(11), 5, 10.1167/14.11.5. 25194017

[bib63] Oriet, C., & Brand, J. (2013). Size averaging of irrelevant stimuli cannot be prevented. *Vision Research,* 79, 8–16, 10.1016/j.visres.2012.12.004. 23274647

[bib64] Parkes, L., Lund, J., Angelucci, A., Solomon, J. A., & Morgan, M. (2001). Compulsory averaging of crowded orientation signals in human vision. *Nature Neuroscience,* 4(7), 739–744, 10.1038/89532. 11426231

[bib65] Pearce, B., Crichton, S., Mackiewicz, M., Finlayson, G. D., & Hurlbert, A. (2014). Chromatic illumination discrimination ability reveals that human colour constancy is optimised for blue daylight illuminations. *PLoS One,* 9(2), e87989, 10.1371/journal.pone.0087989. 24586299 PMC3929610

[bib66] Pelli, D. G. (1997). The videotoolbox software for visual psychophysics: Transforming numbers into movies. *Spatial Vision,* 10, 437–442, 10.1163/156856897X00366. 9176953

[bib67] Rajendran, S., Maule, J., Franklin, A., & Webster, M. A. (2021). Ensemble coding of color and luminance contrast. *Attention, Perception, & Psychophysics,* 83, 911–924, 10.3758/s13414-020-02136-6. PMC8021599 33025468

[bib68] Rosenthal, I., Ratnasingam, S., Haile, T., Eastman, S., Fuller-Deets, J., & Conway, B. R. (2018). Color statistics of objects, and color tuning of object cortex in macaque monkey. *Journal of Vision,* 18(11), 1, 10.1167/18.11.1. PMC6168048 30285103

[bib69] Saarela, T. P., & Landy, M. S. (2012). Combination of texture and color cues in visual segmentation. *Vision Research,* 58, 59–67, 10.1016/j.visres.2012.01.019. 22387319 PMC3448013

[bib70] Tanaka, J. W., & Presnell, L. M. (1999). Color diagnosticity in object recognition. *Perception & Psychophysics,* 61(6), 1140–1153, 10.3758/BF03207619. 10497433

[bib71] Tark, K. J., Kang, M. S., Chong, S. C., & Shim, W. M. (2021). Neural representations of ensemble coding in the occipital and parietal cortices. *NeuroImage,* 245, 118680, 10.1016/j.neuroimage.2021.118680. 34718139

[bib72] Teng, T., Li, S., & Zhang, H. (2021). The virtual loss function in the summary perception of motion and its limited adjustability. *Journal of Vision,* 21(5), 2, 10.1167/jov.21.5.2. PMC8107510 33944907

[bib73] Tyler, C. W., & Solomon, J. A. (2019). Color perception in natural images. *Current Opinion in Behavioral Sciences,* 30, 8–14, 10.1016/j.cobeha.2019.04.002.

[bib74] Utochkin, I. S. (2015). Ensemble summary statistics as a basis for rapid visual categorization. *Journal of Vision,* 15(4), 8, 10.1167/15.4.8. 26317396

[bib75] Utochkin, I. S., Choi, J., & Chong, S. C. (2023). A population response model of ensemble perception. *Psychological Review,* 131(1), 36–57, 10.1037/rev0000426. 37011150

[bib76] Utochkin, I. S., Khvostov, V. A., & Stakina, Y. M. (2018). Continuous to discrete: Ensemble-based segmentation in the perception of multiple feature conjunctions. *Cognition,* 179, 178–191, 10.1016/j.cognition.2018.06.016. 29960219

[bib77] Utochkin, I. S., & Vostrikov, K. O. (2017). The numerosity and mean size of multiple objects are perceived independently and in parallel. *PLoS One,* 12(9), e0185452, 10.1371/journal.pone.0185452. 28957361 PMC5619754

[bib78] Utochkin, I. S., & Yurevich, M. A. (2016). Similarity and heterogeneity effects in visual search are mediated by “segmentability”. *Journal of Experimental Psychology: Human Perception and Performance,* 42(7), 995, 10.1037/xhp0000203. 26784002

[bib79] van Lier, R., &Wagemans, J. (1997). Perceptual grouping measured by color assimilation: Regularity versus proximity. *Acta Psychologica,* 97(1), 37–70, 10.1016/S0001-6918(97)00023-1. 9448513

[bib80] Virtanen, L. S., Olkkonen, M., & Saarela, T. P. (2020). Color ensembles: Sampling and averaging spatial hue distributions. *Journal of Vision,* 20(5), 1, 10.1167/jov.20.5.1. PMC7409613 32392284

[bib81] Wagemans, J., Elder, J. H., Kubovy, M., Palmer, S. E., Peterson, M. A., Singh, M., & Von der Heydt, R. (2012). A century of gestalt psychology in visual perception: I. perceptual grouping and figure–ground organization. *Psychological Bulletin,* 138(6), 1172, 10.1037/a0029333. 22845751 PMC3482144

[bib82] Webster, J., Kay, P., & Webster, M. A. (2014). Perceiving the average hue of color arrays. *Journal of the Optical Society of America A,* 31(4), A283–A292, 10.1364/JOSAA.31.00A283. PMC3979548 24695184

[bib83] Webster, M. A., & Kay, P. (2012). Color categories and color appearance. *Cognition,* 122(3), 375–392, 10.1016/j.cognition.2011.11.008. 22176751 PMC3412132

[bib84] Whitney, D., & Yamanashi Leib, A. (2018). Ensemble perception. *Annual Review of Psychology,* 69, 105–129, 10.1146/annurev-psych-010416-044232. 28892638

[bib85] Wichmann, F. A., Sharpe, L. T., & Gegenfurtner, K. R. (2002). The contributions of color to recognition memory for natural scenes. *Journal of Experimental Psychology: Learning, Memory, and Cognition,* 28(3), 509, 10.1037/0278-7393.28.3.509. 12018503

[bib86] Witzel, C., & Gegenfurtner, K. R. (2011). Is there a lateralized category effect for color? *Journal of Vision,* 11(12), 16, 10.1167/11.12.16. 22019716

[bib87] Witzel, C., & Gegenfurtner, K. R. (2015). Categorical facilitation with equally discriminable colors. *Journal of Vision,* 15(8), 22, 10.1167/15.8.22. 26129860

[bib88] Witzel, C., & Gegenfurtner, K. R. (2018). Color perception: Objects, constancy, and categories. *Annual Review of Vision Science,* 4, 475–499, 10.1146/annurev-vision-091517-034231. 30004833

[bib89] Wolfe, J. M. (2021). Guided search 6.0: An updated model of visual search. *Psychonomic Bulletin & Review,* 28(4), 1060–1092, 10.3758/s13423-020-01859-9. 33547630 PMC8965574

[bib90] Wright, O. (2012). Categorical influences on chromatic search asymmetries. *Visual Cognition,* 20(8), 947–987, 10.1080/13506285.2012.715600.

[bib91] Zhao, Y., Zeng, T., Wang, T., Fang, F., Pan, Y., & Jia, J. (2023). Subcortical encoding of summary statistics in humans. *Cognition,* 234, 105384, 10.1016/j.cognition.2023.105384. 36736077

